# Deep Equatorial Pacific Ocean Oxygenation and Atmospheric CO_2_ Over The Last Ice Age

**DOI:** 10.1038/s41598-020-63628-x

**Published:** 2020-04-20

**Authors:** Franco Marcantonio, Ryan Hostak, Jennifer E. Hertzberg, Matthew W. Schmidt

**Affiliations:** 1 Department of Geology and Geophysics, Texas A&M University, College Station, USA; 2 Department of Earth, Ocean and Atmospheric Sciences, Old Dominion University, Norfolk, USA

**Keywords:** Ocean sciences, Palaeoceanography, Palaeoclimate

## Abstract

Ventilation of carbon stored in the deep ocean is thought to play an important role in atmospheric CO_2_ increases associated with Pleistocene deglaciations. The presence of this respired carbon has been recorded by an array of paleoceanographic proxies from various locations across the global ocean. Here we present a new sediment core from the Eastern Equatorial Pacific (EEP) Ocean spanning the last 180,000 years and reconstruct high-resolution ^230^Th-derived fluxes of ^232^Th and excess barium, along with redox-sensitive uranium concentrations to examine past variations in dust delivery, export productivity, and bottom-water oxygenation, respectively. Our bottom-water oxygenation record is compared to other similar high-resolution records from across the Pacific and in the Southern Ocean. We suggest that the deep Pacific is a site of respired carbon storage associated with periods of decreased global atmospheric CO_2_ concentration during the LGM, confirming the conclusions from a wealth of previous studies. However, our study is the first to show a similar relationship beyond the last glacial, extending to at least 70,000 years.

## Introduction

The dominant signal in global climate over the last 800,000 years is the 100-kyr co-variation of air temperature and atmospheric CO_2_ concentrations observed in the EPICA Antarctica ice core^1^. The temperature variations are likely modulated by the 100-kyr eccentricity signal and amplified by climate feedbacks which involve atmospheric CO_2_. The cause of atmospheric CO_2_ variability over late Pleistocene glacial-interglacial cycles is still debated, with a variety of explanations from changes in ocean stratification and ventilation, efficiency of the biological pump and nutrient availability, and carbonate compensation depth (see^2^ and references therein). One prevalent hypothesis is that there is an increased storage of respired carbon in the deep ocean during glacial maxima that is isolated from interaction with the atmosphere^3–7^. Significant ventilation of this respired carbon pool is thought to occur in the Southern Ocean and is coincident with upwelling there during the deglaciation, and on millennial timescales during the last glacial period^2,4,7^.

Like the Southern Ocean, the eastern equatorial Pacific Ocean (EEP) plays an important role in the global carbon cycle. Today, although the EEP is the major source of oceanic CO_2_ outgassed to the atmosphere^8^, approximately 5–10% of annual global export production occurs there^9^. Thus, although the EEP is a net CO_2_ source to the atmosphere today, it could easily have been a CO_2_ sink in the past, for example, if sequestering of CO_2_ by the biological pump were more efficient^10,11^.

Here we present high-resolution proxy data of bottom water oxygenation (authigenic U, see Methods), biological productivity (^230^Th-derived excess Ba (xsBa) flux, see Methods), and dust (^230^Th-derived ^232^Th flux, see Materials and Methods) flux from a high-sedimentation-rate core collected on the Cocos Ridge bordering the Panama Basin of the EEP (MV1014-8JC (8JC), 6°14.0′N, 86°02.6′W; 1993 m water depth). Our record spans the past 180 kyr beginning in the penultimate glacial period (Marine Oxygen Isotope stage, MIS 6). The sedimentation rate at site 8JC varies from 1.3 to 6.8 cm/kyr, giving a sub-millennial age resolution of ~300–900 yr during MIS 1–4 and a millennial age resolution of ~750–2000 yr during MIS 5 and 6 for our sampling resolution of 2 cm.

Through these records of ^232^Th flux, xsBa flux, and aU concentrations, we aim to better understand the relationship between changes in dust deposition, export production, and bottom water oxygenation in the glacial EEP. We compare our new record with that at the equator (MV1014-17JC^11^) and attempt to unravel the relative contributions of organic matter flux and bottom water oxygen concentrations to pore water redox conditions. We conclude that our EEP high resolution aU records generally reflect changes in bottom water oxygenation that waxes and wanes with the extent and reach of a deep-Pacific respired carbon pool that mirrors variations in atmospheric CO_2_ over the past 180 kyr. However, there are times during which differences between the authigenic uranium records in the EEP may be explained by changes in local export production and/or changes in the depth interval of the respired carbon pool.

## Results and Discussion

### EEP ^232^Th (dust) fluxes

**Figure 1:**
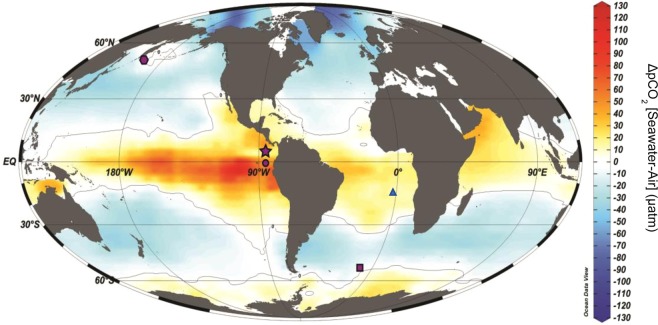
Global ΔpCO_2_ flux and study core locations. Map created in Ocean Data View (Schlitzer, R., version 5.2.0, https://odv.awi.de) using ΔpCO_2_ flux data from Takahashi *et al*. (2009)^8^. Core locations for authigenic uranium records compared in this study are denoted on the map as follows: Subarctic North Pacific site ODP 882 (hexagon), Eastern Equatorial Pacific sites MV1014-8JC (Star) and MV1014-17JC (circle), Southern Ocean sites TN057-13/14PC (square), South Atlantic site TN057-21PC (triangle).

**Figure 2:**
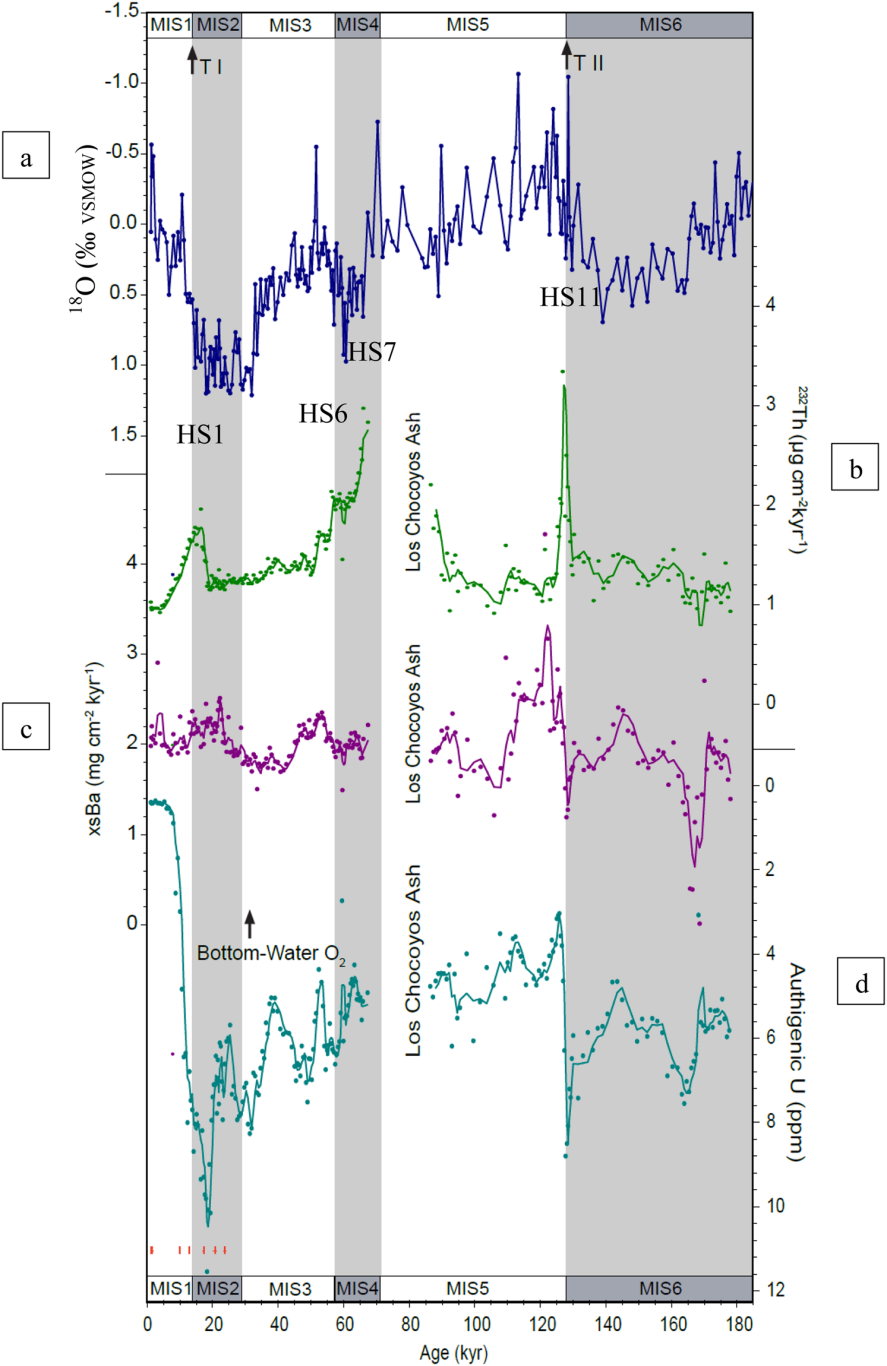
Core MV1014-8JC proxy records. (**a**) δ^18^O record from *N. dutertrei* as ‰VSMOW (dark blue line). (**b)** ^230^Th-normalized ^232^Th flux, a proxy for dust flux (green circles). Green solid line connecting data represents a three-point running mean. Dust flux peaks likely associated with HS1, 6, 7, and 11 are shown. **(c)** ^230^Th-normalized xsBa flux, a proxy for export production (purple circles). Purple solid line connecting data represents a three-point running mean. (**d)** Authigenic uranium, a proxy for bottom-water oxygenation (teal circles). Teal solid line connecting the data represents a three-point running mean. Ages for MIS stage boundaries and Terminations I and II from Lisiecki and Raymo, 2004^45^ (See Methods). Seven radiocarbon ages and uncertainties plotted in red at base of figure (See Methods).

A slowdown of the Atlantic Meridional Overturning Circulation (AMOC)^12^ during the Last Glacial Maximum (LGM) and Heinrich Stadial (HS) 1 is coincident with the shifting of wind belts, specifically the Intertropical Convergence Zone (ITCZ) and the westerlies of both hemispheres^13^. In the EEP, south of site 8JC studied here (core 17JC; Fig. 1, circle), Loveley *et al*.^11^ report evidence of increased pulses of dust (^232^Th fluxes; see Methods section) during the deglaciation and several HS events (climate events initiated in the North Atlantic). These pulses are interpreted to represent a link between changes in atmospheric circulation in the EEP and changes in AMOC. At site 8JC in the EEP (Fig. 1, star), there are significant increases in ^230^Th-derived ^232^Th fluxes at the Terminations of glacial stages (Fig. 2a; see Methods for age model, radiocarbon and δ^18^O stratigraphy). This timing is coincident with the timing of HS1 (~15 kyr, end MIS 2) and HS11 (~129–136 kyr, end MIS 6). Notably, two to three-fold increases in ^230^Th-derived ^232^Th fluxes from “baseline” interglacial values of ~1 µg cm^−2^ kyr^−1^ at HS1 and HS11 indicate a link between periods of deglaciation and increased northern hemisphere dust supplied to the EEP. Within and towards the end of MIS 4, there are two clear dust flux peaks (consistent with the timing of HS6 and HS7) with a similar 2- to 3-fold increase in ^230^Th-derived ^232^Th flux above the baseline values (Fig. 2b). The duration of these four dominant dust flux peaks (HS1, HS6, HS7, HS11) in ^230^Th-derived ^232^Th flux (~5 kyr) agree with other studies that show that the tropical Pacific records peak glacial conditions and a dustier atmosphere within short pulses during and at the end of glacial periods^11,14,15^.

**Figure 3:**
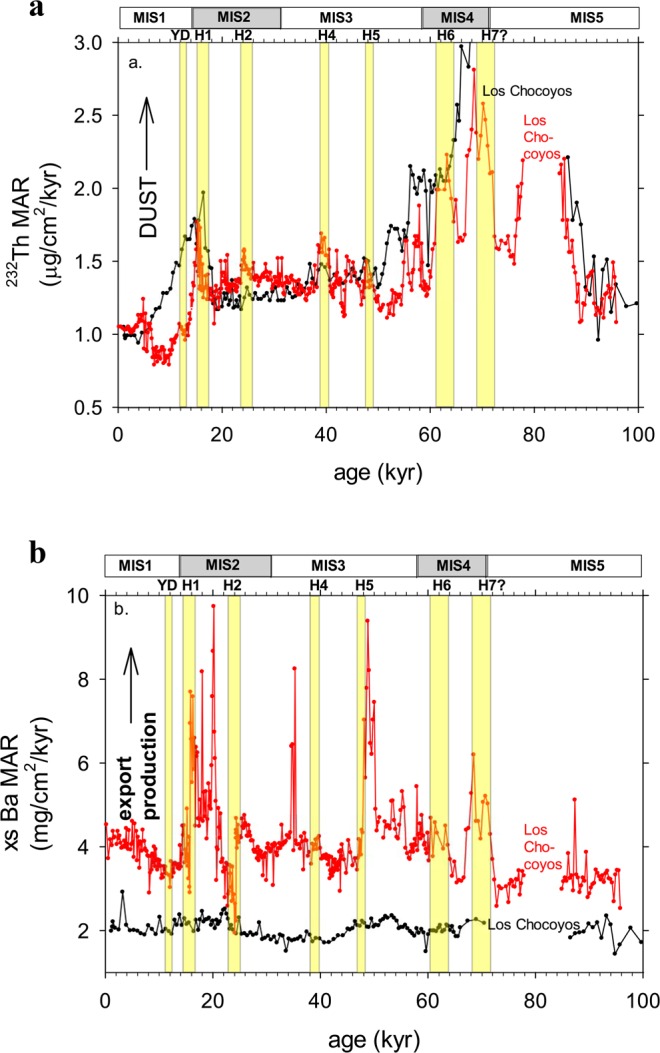
A comparison of proxies for Eastern Equatorial Pacific cores MV1014-8JC (black symbols) and MV1014-17JC (red symbols; Loveley *et al*., 2017^11^) over the past 100 kyr. (**a)** ^232^Th MARs and (**b)** xsBa MARs. Yellow bars represent timing of North Atlantic Heinrich Stadial (HS) events. Data affected by the Los Chocoyos ash are removed (note the greater effect of the ash in 8JC (black) because of bioturbation in this lower sedimentation rate core.

In the easternmost Equatorial Pacific, where dust fluxes are highest because of proximity to the continental margin, we compare the 8JC record with the high-resolution record from 17JC^11^. We are limited to comparing the two dust flux records from 0–70 kyr and 85–95 kyr (Fig. 3a) because the 17JC record spans only the past 95 kyr, and bioturbation of the Los Chocoyos ash obscures the record at 8JC between 70 and 85 kyr. For both cores, during the time intervals of comparison, values for the three highest dust peaks occur during or at the end of MIS 2 and 4, consistent with the timing of HS1, HS6, and HS7. The absolute values of the dust flux peaks in 17JC and 8JC are similar, and are about two to three times those of the baseline values (i.e., ~2–3.5 µg cm^−2^ kyr^−1^ versus ~1–1.3 µg cm^−2^ kyr^−1^ in both cores). Core 17JC clearly records ^230^Th-derived ^232^Th flux variability concurrent with the timing of several other HS events (i.e., HS 2, 4, 5, and 9^11^). These pulses of increased dust flux in 17JC are not prominent in 8JC (HS events cannot be discerned easily in Fig. 3), likely due to the factor-of-two lower sedimentation rate at 8JC which serves to attenuate its record. Note, however, that in the 8JC record (Fig. 3a) there are, for example, several dust flux peaks in MIS3 (e.g., between HS5 and HS6) that may be associated with the pulses identified in 17JC.

At ~6° N, site 8JC is at a position slightly south of the average annual modern-day ITCZ (~7° N^16^). Using the dust flux record from 17JC, it was hypothesized that increases in dust flux, coincident with the timing of several HS events, were likely associated with shifts in the ITCZ, such that the ITCZ was centered on or south of the equator during each HS event^11^. This reasoning was based on the similar ITCZ shifts hypothesized to explain records near or on the South American continent. The similar increases in ^230^Th-derived ^232^Th flux recorded at the more northerly 8JC site at the terminations (HS1 and HS11) and at the end of MIS 4 (HS6) argue that, if changes in the position of the ITCZ are causing the variability, then (1) southern-sourced dust must be dominating the dust delivered to both the 8JC and 17JC sites today and at other times of low dust supply, and (2) northern-sourced dust is being supplied to both sites during times of high dust delivery (consistent with HS events).

### EEP xsBa fluxes

Barium enrichment in ocean sediments occurs at several locations that underlie productive surface waters in the modern ocean^17^. Because barite (BaSO_4_) constitutes most biogenically-produced barium in the ocean, fluxes of barite in marine environments have been widely used as a paleoproductivity proxy (see summary paper by^18^). Excess Ba (xsBa) concentrations, the portion of the total barium sedimentary signal that is produced authigenically (i.e., barite precipitation), can be estimated by subtracting the continentally-derived aluminosilicate Ba from the total sedimentary Ba (see Methods). This paleoproductivity proxy is sensitive to changing pore water redox conditions, such that sulfate-reducing conditions can lead to the remineralization of previously deposited barite and subsequent release of barium into sediment pore waters^19^.

At site 8JC in the EEP, the ^230^Th-derived xsBa flux has an average value of ~2 µg cm^−2^ kyr^−1^ and a low variability (~1.2–2.8 µg cm^−2^ kyr^−1^, Fig. 2c, data in Supporting Information). The 8JC record yields an average ^230^Th-derived xsBa flux approximately half that of site 17JC (average of ~ 4 µg cm^−2^ kyr^−1^^11^,), which is located in the upwelling zone to the south in the high productivity area associated with the Pacific cold tongue region (Fig. 3b). The range of variability in xsBa fluxes at 17JC is also much higher (range of ~2–10 µg cm^−2^ kyr^−1^^11^) than that at 8JC (Fig. 3b). The position of core 17JC beneath the high-productivity waters near the equator is probably the cause of the disparity between the xsBa fluxes at 8JC versus those at 17JC. In addition, the relationship between the xsBa fluxes and dust fluxes at 8JC are not like that at 17JC. At 17JC, there was a relationship between xsBa and dust during or near HS1, 2, 5, 6, and 7, arguing for increased export production because of increased iron fertilization by dust^11,20^. (Note the slight ~1-kyr offset in the xsBa peaks at H1, H2, and H5 in 17JC which has previously been explained as being due to diagenetic affects during the most severe hypoxic conditions^20^, i.e., authigenic uranium values greater than 5–10 ppm^21^). In 8JC, the prominent dust peaks at HS1, 6, and 11 are not associated with peaks in xsBa. The lack of a relationship between productivity and dust flux at 6°N, compared to that which is observed at the equator (17JC; Loveley *et al*.^11^), suggests that the increased productivity is a local effect at work only within the upwelling zone at the equator. Equatorial upwelling at 17JC (not existent at 8JC at 6°N) yields an abundant supply of nutrients to this high-nutrient, low-chlorophyll (HNLC) zone that is limited by the micronutrient, Fe. Only upon the relaxation of this Fe limitation by increased dust delivery can productivity be enhanced. Although the idea for a connection between millennial dust delivery and productivity in the eastern equatorial is controversial^20–22^, there is recent evidence suggesting that Fe-induced stimulation of nitrogen fixation can cause significant increases in export production^23^.

### Authigenic uranium and deep ocean carbon storage in the EEP

**Figure 4:**
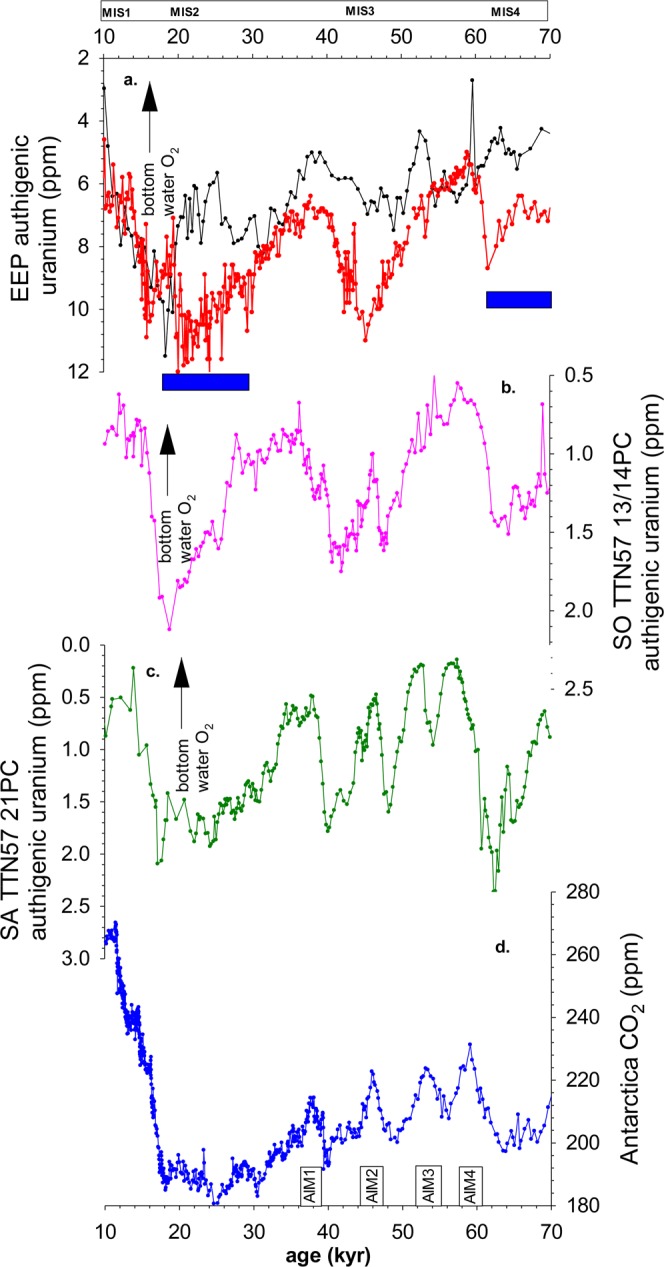
(**a**) Authigenic U records from the eastern equatorial Pacific Ocean (MV1014-8JC, black circles; MV1014-17JC, red circles). Blue bars represent times of coincident high aU and low CO_2_ concentrations in 17JC, 13/14PC, and 21PC. (**b)** Authigenic U composite record from cores TN057-13PC (10–20 kyr) and TN057-14PC (20–70 kyr) from the Southern Ocean (pink circles, Jaccard *et al*., 2016^2^). (**c)** Authigenic U record from core TN057-21PC from the South Atlantic Ocean (green circles, authigenic U data from Sachs and Anderson, 2003^38^; age model from Gottschalk *et al*., 2016^7^). (**d)** Atmospheric CO_2_ record from Antarctic ice cores (blue circles; Bereiter *et al*., 2012^1^). Southern Hemisphere Antarctic Isotope Maxima (AIM) 1–4 are shown.

Most important in our work here is the identification of a decoupling between the ^232^Th/xsBa and the authigenic uranium signals in cores 8JC and 17JC. There is no apparent or systematic relationship with HS events, for example, in either of the cores. This decoupling is clearly detected when comparing the range in values of each proxy at 8JC versus that at 17JC. Specifically, while there is a significant three- to four-fold increase in the range of the ^230^Th-derived xsBa fluxes at site 17JC compared to that in 8JC (Fig. 3b), there is not a similar disparity between the range of aU concentrations recorded in the two cores (Fig. 4a). Indeed, the range in aU concentrations, excluding the Holocene part of the record, is from 4–12 ppm in both 8JC and 17JC (Fig. 4a; data in Supporting Information). There are some significant temporal differences between the authigenic uranium values (Fig. 4), which we describe later, but the range in both cores is the same.

Authigenic U concentrations can be a function of either the flux of reducible organic matter to the sediment^24–26^ and/or the oxygen concentrations of the overlying bottom water^27–29^. Given that the flux of reducible organic matter (i.e., the xsBa flux proxy) is significantly different at both sites, while the range in authigenic U concentrations is similar at both sites, leads us to conclude that the high aU concentrations at both sites are controlled by the low oxygen concentrations of overlying bottom waters. If the sedimentary flux of organic matter (which is higher in the zone of upwelling at 17JC) were controlling the aU concentrations in 8JC, one would expect the aU concentrations in 8JC to be significantly lower than those in 17JC. At the two sites, separated by 650 km, the general inconsistency of the productivity signals (xsBa flux, Fig. 3) versus the similar range in the authigenic U signal (Fig. 4) suggests that bottom water oxygenation, and the potential for a widespread deep respired carbon pool, is a regional feature that dominates the authigenic U record. There is no question, however, that superimposed on this “baseline,” there are various intervals (during the deglaciation and mid-MIS3) during which local changes in export production influence the authigenic U signal, which may be responding to the flux of raining reducible organic matter (i.e., export production fluxes and authigenic U concentrations vary in concert). We discuss this further below.

Substantial evidence for an increased size of the respired carbon pool in the EEP at the LGM exists in the form of observations of the deepening of oxygen-depleted waters^30–34^. Our two high-resolution aU records, one from ~6° N at 1993 m water depth (8JC, this study), and the other on the equator at 2846 m water depth (17JC[^11^), expands our understanding of the deepening of oxygen-depleted waters in the EEP beyond timescales of previous studies to the past 70 kyr.

Authigenic uranium concentrations at 8JC are highest during MIS 2 and 6 (Figs. 2d and 4a). Similarly, aU concentrations at 17JC are highest during MIS 2 (Fig. 4a^11^, no data for MIS 6). We hypothesize that at these times when aU concentrations are the highest in both cores (Fig. 4a), bottom-water oxygen concentrations are the lowest and may represent an increased extent of a respired carbon pool in the deep equatorial Pacific Ocean. However, there are specific instances within glacial times during when the records diverge. The disparities between these EEP aU records occur for ~5–10 kyr during MIS 4 and early MIS 2 (Fig. 4a). There are slight offsets during MIS3 from 38–54 kyr that are likely within the errors of both age models. Differences in the aU concentrations and trends at the two sites appear to be greatest at times when the Antarctic ice core record shows pronounced and prolonged episodes of low CO_2_ (180–200 ppm during MIS 2 and MIS 4; Fig. 4). The differences during these times may be due to the difference in depth between the two sites. At 2846 m water depth, core 17JC in the Panama Basin is well within the expected lower 2-km depth range associated with glacial increases in deep Pacific respired carbon storage^33–37^. However, off-equatorial site 8JC (1993 m) is slightly above the 2-km depth estimate for deep waters which show an increased glacial respired carbon storage. In this context, it is possible that the decreases in aU values at site 8JC at the same time that 17JC shows enhancement result from changes in the depth of the respired carbon pool in the EEP. That is, the site depths are telling us that, during the times of lowest CO_2_ concentrations, the location of the respired carbon pool moves to greater depth, and the zone of deoxygenation expands proportionally greater in the downward direction.

Importantly, the most significant decreases in aU occur at the glacial terminations, i.e., at the ends of MIS 2 (8JC and 17JC, Fig. 4a) and 6 (8JC, Fig. 2d), and likely reflect an increase in bottom water oxygen concentrations, as would be expected with a reinvigoration of deep-water ventilation. We suggest that this increased ventilation may release to the atmosphere a portion of the deep respired carbon pool, thereby increasing atmospheric CO_2_ concentrations.

### Global changes in glacial-interglacial respired carbon storage and atmospheric CO_2_

Increased deep ocean respired carbon storage during the LGM, inferred using authigenic uranium concentrations, which are sensitive to changes in bottom water oxygen concentrations, has been demonstrated in the Southern^2^, South Atlantic^7,38^, and Pacific Oceans^11,30,35,36,39^ (Fig. 4). Increases in aU concentrations in each of these records during the most prolonged periods of low glacial atmospheric CO_2_ levels have been hypothesized to represent decreases in bottom water oxygen concentrations and, by inference, increased storage of respired carbon in the deep ocean at these locations.

Records from cores 8JC and 17JC present a case for multiple, repeated periods of deep ocean respired carbon storage in the EEP (previous section). There is some similarity between our records and other high-resolution, high-sedimentation-rate records from the Southern Ocean^2^ (TTN57-13/14PC; water depth 3700 m; Fig. 1 purple square) and the South Atlantic (TTN57-21PC; water depth 4981 m; Fig. 1 blue triangle)^7,38^ in Fig. 4. Specifically, the EEP, Southern Ocean, and South Atlantic aU records each demonstrate a low-frequency variability (~10–20 kyr) in aU concentrations from late MIS 4 to peak glaciation (MIS 2) (Fig. 4b–d). Note that the aU concentrations in the 8JC and 17JC EEP cores are more than 5 times higher than the highest aU concentrations observed in either TTN57-13/14PC or TTN57-21PC (Fig. 4), and is likely due to the equatorial location of these sites that have increased organic rain that might be better preserved through the shallower water column. However, the pattern of variability in the aU records from cores 8JC, 17JC, TTN57-13/14PC, and TN57-21 is such that since MIS 4, there is a general consistency between the records and the Antarctic CO_2_ ice core record (Fig. 4).

The South Atlantic, Southern Ocean and 17JC records have among their highest aU concentrations (lowest bottom water O_2_) during MIS2 and MIS4 (horizontal blue bars shown in Fig. 4a), when atmospheric CO_2_ is less than 200 ppm. This is not the case for the 8JC record as described above. During MIS3 when atmospheric CO_2_ concentrations are relatively higher during the Antarctic Isotope Maxima (AIM) 1–4 (Fig. 4d), the Southern Ocean and South Atlantic cores, within error of the age models of each, have the lowest aU concentrations (highest bottom water O_2_ concentrations (Fig. 4). This is what lead both Jaccard *et al*. (2016)^2^ and Gottschalk *et al*. (2016)^7^ to suggest that growth and shrinking of the respired carbon pool (as manifest by the qualitative indicator of authigenic U concentrations and deep ocean oxygenation) is associated with decreased and increased global atmospheric CO_2_ concentrations, respectively. During MIS3 at both EEP cores there is the suggestion of higher bottom water oxygenation during relative highs in the CO_2_ record (AIM 1, 3, and 4). However, during mid-MIS3, between about 45–48 kyr (AIM2 with relatively high atmospheric CO_2_) in both cores 17JC and 8JC, there is a clear increase in aU which is the opposite of what is observed for both the Southern Ocean and South Atlantic cores. The aU peak in both EEP cores is associated with relatively increased xsBa fluxes (Fig. 3), suggesting the influence of increased export production which may, therefore, also be playing a role in influencing the aU signal. The timing of the aU peaks in MIS3 is coincident with a significant increase in opal fluxes across the easternmost equatorial Pacific^40^, and suggests increased diatom production during a period of enhanced upwelling and strengthened winds. There is also evidence for increased dust fluxes in the easternmost EEP during this time (Fig. 3). Similarly, there is a peak in opal fluxes (diatom production) across the EEP during the deglaciation^40^ when we also observe a mismatch between the 17JC aU record (HS1; along with both increased dust and export production fluxes, Fig. 3) and the increasing atmospheric CO_2_ concentrations (Fig. 4).

Therefore, in the EEP the aU archives are recording a changing bottom water oxygenation pattern that is suggestive of coherent increases and decreases in long-term storage of respired carbon from ~70 kyr up to the end of MIS 2. This pattern is similar, but not identical, to that observed in the Southern and South Atlantic Ocean cores, which is thought to be associated with changing atmospheric CO_2_ concentrations. We believe that both EEP records reflect a general pattern of changing bottom water oxygenation concentrations and base this argument on the similarity in the range of the extremely high authigenic U concentrations at two sites that are vastly different in terms of the flux of organic matter reaching the seafloor. There is a caveat, however: there are times (mid-MIS3 and HS1) during which the EEP authigenic U records are influenced by increased export production fluxes and do not, therefore, reflect changes in a global respired carbon pool associated with those in global atmospheric CO_2_ concentrations. Although our aU results, along with others^11,30,35,36,39,41^, emphasize the importance of the deep Pacific Ocean (along with the deep Southern Ocean and the deep South Atlantic Ocean) as being a potential location for storage of respired carbon during periods of decreased global atmospheric CO_2_ concentrations, it is clear, given our caveats, that further studies using bottom water oxygenation proxies in conjunction with those for organic matter flux are needed to decipher the global extent of deep respired carbon storage.

## Methods

### Analytical

Oxygen isotope analyses were performed using a Thermo Electron Kiel IV sample preparation instrument with an attached Thermo MAT 253 stable isotope ratio mass spectrometer on samples of *N. dutertrei* (>250 μm), and took place at the Stable Isotope Geoscience Facilities (SIGF) of Texas A&M University. Isotope values are reported in delta notation (Supporting Information Table 1) relative to the Vienna Standard Mean Ocean Water isotopic standard.

Seven radiocarbon analyses were performed on the first 100 cm of core MV1014-8JC on the planktonic foraminifer *Neogloboquadrina dutertrei* (>250 μm), and are reported in Supporting Information Table 2. Analyses were performed at the NOSAMS facility at the Woods Hole Oceanographic Institute. Radiocarbon ages were calibrated to calendar age using Calib 7.0 Marine13^42,43^ with a 400-yr reservoir age correction.

Sediments for MV1014-8JC were sampled approximately every 2 cm and analyzed for uranium, thorium, and barium isotopes using inductively coupled plasma mass spectrometry (ICP-MS) on a magnetic sector Element XR at Texas A&M University. All data appear in the Supporting Information Table 1. To prepare samples for isotope dilution analysis, 0.3–0.4 g of sediment was spiked with ^229^Th and ^236^U and then digested in a cocktail of HNO_3_, HClO_4_ and HF. Following complete sediment digestion, an aliquot was removed, diluted, and spiked with ^135^Ba for separate analysis. Samples for U-Th analysis were then further processed through Fe-oxyhydroxide coprecipitation and subsequent anion exchange chromatography in order to separate the Th and U. The National Institute of Standards and Technology Uranium 500 Standard (NIST U500) was used to correct for instrumental mass bias and was analyzed multiple times within each of batch of samples for which Th and U was measured.

### Age models

We constructed a preliminary age model using 1) seven radiocarbon dates on *N. dutertrei* between 0 and 100 cm depth in core, 2) the Los Chocoyos ash (84 kyr)^44^ identified at 311 cm in the core, and 3) tying the 8JC δ^18^O record (*N. dutertrei*) to the LRO4 global oxygen isotope record^45^ via the software Analyseries^46^. The stage boundaries between MIS 4 and 5, and MIS 5 and 6 were the most difficult to discern, given the millennial structure in the oxygen isotope data. The age model for core MV1014-17JC was further refined from that in Loveley *et al*.^11^. Given the strong correlation between the ^230^Th mass accumulation rate (MAR) and Heinrich Stadial (HS) events in 17JC, the age model was refined by tuning the additional millennial scale ^230^Th MAR cycles between HS events to the NGRIP ice core record from 30 kyr to 95 kyr.

### ^230^Th Normalization

The ability to accurately determine past sedimentary fluxes is crucial to reliable paleoclimate reconstructions. ^230^Th is a particle reactive isotope and has a comparatively short residence time relative to its parent isotope ^234^U (tens of years versus 200–400 kyr)^47,48^. Scavenging of aqueous ^230^Th by settling particles leads to its ultimate deposition in marine sediments. The assumption that ^230^Th flux represents its production rate in the water column allows for the determination of sedimentary MARs. Important to the ^230^Th flux method is the fact that it allows for differentiation between the vertical flux form the overlying water column and the flux of sediments redistributed by bottom water currents. The latter flux can be discerned by calculating ^230^Th-derived sediment focusing factors (Ψ)^49^. The utility of focusing factors lies in their ability to measure periods of focusing and winnowing in different oceanic sedimentary environments.

^230^Th is preferentially scavenged by fine grain particles because they have a higher surface area to volume ratio^50^. There may exist, therefore, a biasing effect on the measured activities of ^230^Th in areas that have also been subjected to grain size biasing during redistribution processes. This would ultimately affect ^230^Th derived MARs as well. The potential biasing of ^230^Th-normalized sediment fluxes at highly focused Atlantic sites was modeled with an aim to correct for syndepositional grain size biasing^50^. It was determined that only sites with focusing factors (Ψ) higher than 5.9 showed significant biasing of the ^230^Th-derived MARs. Within the Panama Basin, previous studies show that there is no more than a 30% biasing of ^230^Th-normalized MARs when *Ψ* is less than about 4^51^. Average focusing factors for the interval of sediment deposited within each oxygen-isotope stage (MIS 1 through 6) are consistently less than 4. The highest average focusing factor is 4.5 during MIS 2. Furthermore, xs^230^Th-normalized accumulation rates of proxies that are contained predominantly in the fine-grained fraction of the sediment (such as the ^232^Th, xsBa and authigenic U proxies used here) are not significantly sensitive to the ^230^Th fractionation observed during sediment redistribution processes, which seems to affect coarse-grained component accumulation rates to a greater extent^52^. Therefore, accumulation rates for proxies that are virtually entirely contained within the fine-grained component of the sediment can be approximated by multiplying the concentration of the proxy with the ^230^Th-derived MAR.

### ^232^Th Flux

Windblown dust is primarily supplied to ocean sediments from continental material, which has an average ^232^Th crustal concentration of ∼14 ppm^53^. The fraction of dust in sediment samples can, therefore, be estimated by dividing the sample ^232^Th concentration by 14 ppm. Numerous studies in the past have used ^232^Th as a proxy for windblown dust in the equatorial Pacific Ocean (see^11^, and references therein) in this way. We prefer to give the raw ^232^Th fluxes here rather than converting to dust fluxes so that the reader can more easily compare to previous studies in the literature which use different average dust ^232^Th concentration.

### xsBa Flux

The association between biogenic “excess” Ba (xsBa) delivered to ocean sediment as the mineral barite^17^, and organic matter permits the use of xsBa fluxes as a proxy for past primary productivity^17^ timescales. To calculate the xsBa concentration in sediment samples, the detrital Ba component must be subtracted. This detrital, or lithogenic component is estimated by multiplying the detrital Th concentration by the average upper crustal Ba/Th ratio of 51.4^54^. Because barite is primarily found contained in the sediment fine-grained fraction of <5 μm^17^, ^230^Th-derived xsBa fluxes are likely not biased by syndepositional sediment redistribution^52^.

### Authigenic U Concentrations

Uranium is supplied to the sediments by the deposition of organic matter, sediment pore water redox conditions, and diffusion from higher concentrations in bottom waters to lower concentrations in pore waters^55^. Dissolved uranium present in pore waters exhibits similar redox behavior to iron. Near the sediment sub-oxic to anoxic boundary, iron is reduced from Fe(III) to Fe(II), and uranium, present as the highly soluble UO_2_(CO_3_)_3_^4−^ complex, is reduced from U(VI) to U(IV), leading to the precipitation of authigenic uranium, presumably as insoluble uranium oxide^56^. Authigenic uranium enrichment can, therefore, be produced by high rates of organic carbon deposition^55^, and/or changes in bottom water oxygenation^57^. Moffitt *et al*.^21^ estimate that authigenic U values greater than 5–10 ppm (such as those observed here in the EEP) represent times of severe hypoxia ([O_2_ concentrations < 0.5 mL/L).

## Supplementary information

Supplementary information.

## Data Availability

U, Th, Ba, and oxygen data will be archived at the National Oceanic and Atmospheric Administration National Centers for Environmental Information (NCEI) database upon publication and are also available as a supplement to this manuscript.
