# Supplementary material for: Deep Equatorial Pacific Ocean Oxygenation and Atmospheric CO2 Over The Last Ice Age
Source: Sci Rep. 2020 Apr 20;10:6606. doi: 10.1038/s41598-020-63628-x (PMC7171191; doi:10.1038/s41598-020-63628-x)
Supplement: Supplementary file 1 — Supplementary information. [file 41598_2020_63628_MOESM1_ESM.docx]

**DEEP EQUATORIAL PACIFIC OCEAN OXYGENATION AND ATMOSPHERIC CO_2_ OVER THE LAST ICE AGE**

Franco Marcantonio^1,*^ (marcantonio@tamu.edu), Ryan Hostak^1^ (r.hostak@gmail.com), Jennifer E. Hertzberg^2^ ([Jennifer.Hertzberg@gmail.com](mailto:Jennifer.Hertzberg@gmail.com)), Matthew W. Schmidt^2^ (mwschmid@odu.edu)

1. Department of Geology and Geophysics, Texas A&M University
2. Department of Earth, Ocean and Atmospheric Sciences, Old Dominion University

*corresponding author

**Supplemental Tables File**

**Supplemental Table 1**

**MV1014-8JC, 6° 14.0’ N, 86° 02.6’ W; 1993 m water depth**

|  |  |  |  |  |  |  |  |
| --- | --- | --- | --- | --- | --- | --- | --- |
|  |  |  |  |  |  |  |  |
|  | **Depth (cm)** | **Age Model (kyr)** | **^230^Th-derived MAR (g cm^-2^ kyr^-1^)** | **^230^Th-derived ^232^Th MAR (μg cm^-2^ kyr^-1^)** | **Authigenic U (ppm)** | **^230^Th-derived xsBa MAR (mg cm^-2^ kyr^-1^)** | **δ^18^O _(VSMOW)_** |
|  | 1 | 1.1 | 0.75 | 1.05 | 0.38 | 1.99 | 0.05 |
|  | 3 | 1.3 | 0.69 | 0.99 | 0.40 | 2.09 | -0.57 |
|  | 5 | 1.4 | 0.74 | 0.97 | 0.40 | 2.21 | -0.34 |
|  | 7 | 1.8 | 0.71 | 0.99 | 0.39 | 2.06 | -0.48 |
|  | 9 | 2.5 | 0.69 | 0.99 | 0.34 | 2.02 | 0.11 |
|  | 11 | 3.2 | 0.69 | 0.99 | 0.40 | 2.92 | 0.25 |
|  | 13 | 3.9 | 0.70 | 0.94 | 0.40 | 2.13 | -0.03 |
|  | 15 | 4.6 | 0.72 | 1.01 | 0.42 | 2.00 | 0.03 |
|  | 17 | 5.3 | 0.73 | 1.02 | 0.36 | 2.01 | 0.06 |
|  | 19 | 6.0 | 0.86 | 1.06 | 0.52 | 1.99 | 0.13 |
|  | 21 | 6.6 | 0.74 | 1.12 | 0.52 | 1.93 | 0.50 |
|  | 23 | 7.3 | 0.73 | 1.17 | 0.63 | 1.90 | 0.30 |
|  | 25 | 8.0 | 0.78 | 1.18 | 0.87 | 2.12 | 0.08 |
|  | 27 | 8.7 | 0.78 | 1.28 | 2.53 | 2.03 | 0.29 |
|  | 29 | 9.4 | 0.78 | 1.28 | 1.70 | 1.91 | 0.05 |
|  | 31 | 10.1 | 1.28 | 1.31 | 2.97 | 2.32 | 0.25 |
|  | 33 | 10.6 | 0.86 | 1.42 | 4.81 | 1.97 | -0.21 |
|  | 35 | 11.2 | 0.91 | 1.49 | 6.41 | 2.04 | 0.11 |
|  | 37 | 11.8 | 0.86 | 1.47 | 6.33 | 1.98 | 0.49 |
|  | 39 | 12.3 | 0.88 | 1.58 | 7.97 | 1.92 | 0.55 |
|  | 41 | 12.9 | 0.95 | 1.67 | 6.76 | 2.25 | 0.49 |
|  | 43 | 13.3 | 0.91 | 1.65 | 7.45 | 2.13 | 0.55 |
|  | 45 | 13.8 | 0.96 | 1.65 | 7.67 | 2.38 | 0.53 |
|  | 47 | 14.2 | 0.94 | 1.74 | 8.66 | 2.13 | 0.70 |
|  | 49 | 14.7 | 1.01 | 1.79 | 8.01 | 2.29 | 1.02 |
|  | 51 | 15.1 | 0.94 | 1.62 | 7.77 | 2.15 | 0.61 |
|  | 53 | 15.5 | 0.98 | 1.78 | 8.04 | 2.19 | 0.94 |
|  | 57 | 16.4 | 0.92 | 1.97 | 9.31 | 1.98 | 0.97 |
|  | 59 | 16.8 | 1.02 | 1.59 | 8.16 | 2.25 | 0.78 |
|  | 61 | 17.3 | 0.89 | 1.57 | 9.26 | 2.01 | 0.68 |
|  | 63 | 17.6 | 0.99 | 1.46 | 9.68 | 2.26 | 0.89 |
|  | 65 | 18.0 | 1.07 | 1.45 | 9.77 | 2.46 | 1.20 |
|  | 67 | 18.3 | 0.86 | 1.21 | 11.50 | 2.21 | 1.08 |
|  | 69 | 18.7 | 0.92 | 1.24 | 10.04 | 2.26 | 1.19 |
|  | 71 | 19.0 | 0.92 | 1.17 | 8.96 | 2.27 | 0.95 |
|  | 73 | 19.3 | 0.92 | 1.17 | 10.11 | 2.30 | 0.87 |
|  | 75 | 19.7 | 0.94 | 1.31 | 7.92 | 2.13 | 0.96 |
|  | 77 | 20.0 | 0.88 | 1.19 | 7.36 | 2.23 | 1.06 |
|  | 79 | 20.4 | 0.95 | 1.28 | 7.07 | 2.05 | 0.89 |
|  | 81 | 20.7 | 0.92 | 1.24 | 7.08 | 2.25 | 1.14 |
|  | 83 | 21.0 | 0.89 | 1.28 | 6.39 | 2.15 | 0.80 |
|  | 85 | 21.3 | 0.89 | 1.20 | 7.75 | 2.21 | 0.94 |
|  | 87 | 21.6 | 1.07 | 1.23 | 6.49 | 2.39 | 0.95 |
|  | 89 | 21.9 | 1.07 | 1.37 | 7.53 | 2.49 | 0.68 |
|  | 91 | 22.2 | 1.06 | 1.18 | 6.08 | 2.52 | 0.88 |
|  | 93 | 22.5 | 1.07 | 1.19 | 6.16 | 2.40 | 1.15 |
|  | 95 | 22.8 | 0.93 | 1.23 | 7.00 | 2.28 | 1.11 |
|  | 97 | 23.1 | 0.85 | 1.19 | 7.90 | 2.04 | 1.06 |
|  | 99 | 23.4 | 0.90 | 1.26 | 7.21 | 2.13 | 1.13 |
|  | 101 | 23.7 | 0.89 | 1.17 | 6.58 | 2.02 | 0.94 |
|  | 103 | 24.3 | 0.87 | 1.25 | 6.05 | 1.93 | 1.06 |
|  | 105 | 24.8 | 1.00 | 1.32 | 5.97 | 2.31 | 1.18 |
|  | 107 | 25.3 | 0.88 | 1.27 | 5.66 | 1.92 | 1.20 |
|  | 109 | 25.9 | 0.82 | 1.24 | 7.30 | 1.93 | 1.14 |
|  | 111 | 26.4 | 0.87 | 1.28 | 7.11 | 1.88 | 0.90 |
|  | 113 | 27.0 | 0.89 | 1.24 | 7.39 | 1.95 | 0.76 |
|  | 115 | 27.5 | 0.79 | 1.24 | 7.91 | 1.89 | 0.91 |
|  | 117 | 28.1 | 0.90 | 1.26 | 7.82 | 1.92 | 0.81 |
|  | 119 | 28.6 | 1.08 | 1.28 | 7.76 | 2.19 | 1.13 |
|  | 121 | 29.2 | 0.86 | 1.23 | 7.48 | 1.79 | 1.17 |
|  | 123 | 29.7 | 0.87 | 1.29 | 7.13 | 1.82 | 1.10 |
|  | 125 | 30.3 | 0.84 | 1.32 | 7.04 | 1.87 | 1.02 |
|  | 127 | 30.8 | 0.81 | 1.23 | 8.01 | 1.78 | 1.04 |
|  | 129 | 31.4 | 0.83 | 1.23 | 8.23 | 1.85 | 1.03 |
|  | 131 | 31.9 | 0.86 | 1.23 | 8.11 | 1.71 | 1.21 |
|  | 133 | 32.5 | 0.92 | 1.32 | 6.80 | 1.90 | 0.91 |
|  | 135 | 33.0 | 0.89 | 1.28 | 6.86 | 1.87 | 0.42 |
|  | 137 | 33.6 | 0.82 | 1.25 | 7.26 | 1.51 | 0.92 |
|  | 139 | 34.1 | 0.83 | 1.26 | 7.31 | 1.80 | 0.63 |
|  | 141 | 34.7 | 0.91 | 1.34 | 6.68 | 1.76 | 0.39 |
|  | 143 | 35.2 | 0.92 | 1.28 | 6.28 | 1.78 | 0.64 |
|  | 145 | 35.8 | 0.91 | 1.31 | 6.45 | 1.80 | 0.58 |
|  | 147 | 36.3 | 0.94 | 1.35 | 5.60 | 1.85 | 0.39 |
|  | 149 | 36.9 | 0.90 | 1.48 | 5.86 | 1.75 | 0.60 |
|  | 151 | 37.4 | 1.02 | 1.38 | 5.15 | 1.97 | 0.37 |
|  | 153 | 38.0 | 0.96 | 1.34 | 5.01 | 1.90 | 0.43 |
|  | 155 | 38.5 | 0.92 | 1.44 | 5.32 | 1.74 | 0.31 |
|  | 157 | 39.1 | 0.92 | 1.48 | 5.02 | 1.82 | 0.67 |
|  | 159 | 39.8 | 0.96 | 1.46 | 5.33 | 1.81 | 0.55 |
|  | 161 | 40.7 | 0.88 | 1.37 | 5.75 | 1.71 | 0.38 |
|  | 163 | 41.6 | 0.88 | 1.41 | 5.87 | 1.72 | 0.50 |
|  | 165 | 42.5 | 0.86 | 1.34 | 5.83 | 1.79 | 0.35 |
|  | 167 | 43.3 | 0.89 | 1.35 | 5.85 | 1.88 | 0.40 |
|  | 169 | 44.2 | 0.90 | 1.41 | 6.18 | 1.88 | 0.15 |
|  | 171 | 45.1 | 0.90 | 1.45 | 6.64 | 2.04 | 0.06 |
|  | 173 | 45.5 | 0.86 | 1.40 | 6.98 | 2.02 | 0.36 |
|  | 175 | 46.0 | 0.88 | 1.37 | 6.57 | 2.05 | 0.44 |
|  | 177 | 46.4 | 0.88 | 1.38 | 6.61 | 2.22 | 0.32 |
|  | 179 | 46.8 | 0.89 | 1.43 | 6.87 | 2.13 | 0.39 |
|  | 181 | 47.3 | 0.95 | 1.52 | 6.16 | 2.11 | 0.16 |
|  | 183 | 47.7 | 0.94 | 1.51 | 6.42 | 2.23 | 0.32 |
|  | 185 | 48.1 | 0.92 | 1.50 | 6.41 | 2.18 | 0.42 |
|  | 187 | 48.6 | 0.88 | 1.37 | 7.02 | 2.09 | 0.36 |
|  | 189 | 49.0 | 0.86 | 1.41 | 7.49 | 2.08 | 0.47 |
|  | 191 | 49.5 | 0.95 | 1.45 | 6.46 | 2.28 | 0.45 |
|  | 193 | 49.9 | 0.89 | 1.32 | 6.46 | 2.09 | 0.16 |
|  | 195 | 50.3 | 0.86 | 1.34 | 6.95 | 2.13 | 0.34 |
|  | 197 | 50.8 | 0.88 | 1.41 | 6.24 | 2.12 | 0.12 |
|  | 199 | 51.2 | 0.92 | 1.48 | 5.57 | 2.23 | -0.02 |
|  | 201 | 51.7 | 1.01 | 1.62 | 5.38 | 2.27 | -0.55 |
|  | 203 | 52.1 | 1.03 | 1.70 | 4.86 | 2.34 | 0.20 |
|  | 205 | 52.5 | 1.06 | 1.74 | 4.34 | 2.30 | 0.32 |
|  | 209 | 53.3 | 1.07 | 1.72 | 4.64 | 2.36 | 0.14 |
|  | 211 | 53.7 | 0.97 | 1.72 | 5.21 | 2.30 | 0.21 |
|  | 213 | 54.2 | 0.94 | 1.65 | 6.16 | 2.23 | 0.02 |
|  | 215 | 54.6 | 0.85 | 1.57 | 6.72 | 2.05 | 0.13 |
|  | 217 | 55.0 | 0.89 | 1.71 | 6.25 | 2.08 | 0.29 |
|  | 219 | 55.4 | 0.88 | 1.66 | 6.14 | 2.08 | 0.19 |
|  | 221 | 55.8 | 0.93 | 1.76 | 5.62 | 2.13 | 0.28 |
|  | 223 | 56.2 | 0.89 | 2.15 | 6.10 | 2.05 | 0.47 |
|  | 225 | 56.7 | 0.89 | 2.09 | 6.24 | 1.98 | 0.32 |
|  | 227 | 57.1 | 0.83 | 2.00 | 6.30 | 1.93 | 0.71 |
|  | 229 | 57.5 | 0.80 | 1.96 | 6.59 | 1.87 | 0.19 |
|  | 231 | 57.9 | 0.84 | 2.07 | 6.35 | 2.05 | 0.13 |
|  | 233 | 58.3 | 0.82 | 2.05 | 6.18 | 1.94 | 0.50 |
|  | 235 | 58.8 | 0.82 | 2.12 | 6.05 | 2.03 | 0.49 |
|  | 237 | 59.2 | 0.80 | 1.98 | 5.37 | 1.95 | 0.23 |
|  | 239 | 59.6 | 0.62 | 1.47 | 2.71 | 1.50 | 0.45 |
|  | 241 | 60.0 | 0.79 | 2.05 | 6.04 | 1.91 | 0.92 |
|  | 243 | 60.4 | 0.73 | 1.97 | 5.49 | 2.00 | 0.56 |
|  | 245 | 60.8 | 0.77 | 2.02 | 5.44 | 2.00 | 0.97 |
|  | 247 | 61.1 | 0.75 | 2.09 | 5.44 | 2.00 | 0.69 |
|  | 249 | 61.5 | 0.73 | 1.99 | 5.19 | 1.98 | 0.49 |
|  | 251 | 61.9 | 0.80 | 2.13 | 4.95 | 2.06 | 0.32 |
|  | 253 | 62.2 | 0.78 | 2.08 | 4.67 | 2.13 | 0.54 |
|  | 255 | 62.6 | 0.74 | 2.05 | 4.57 | 1.97 | 0.64 |
|  | 257 | 63.0 | 0.76 | 2.05 | 4.72 | 2.01 | 0.31 |
|  | 259 | 63.4 | 0.77 | 2.13 | 4.23 | 2.14 | 0.37 |
|  | 261 | 63.7 | 0.78 | 2.15 | 4.62 | 2.11 | 0.45 |
|  | 263 | 64.1 | 0.77 | 2.22 | 5.05 | 2.01 | 0.60 |
|  | 265 | 64.5 | 0.81 | 2.33 | 4.91 | 2.10 | 0.41 |
|  | 267 | 64.8 | 0.75 | 2.33 | 5.07 | 1.96 | 0.40 |
|  | 269 | 65.2 | 0.87 | 2.57 | 5.00 | 1.86 | 0.41 |
|  | 271 | 65.6 | 0.77 | 2.46 | 5.54 | 1.86 | 0.37 |
|  | 273 | 66.0 | 0.92 | 2.97 | 5.10 | 2.07 | 0.65 |
|  | 275 | 67.4 | 0.93 | 2.83 | 4.89 | 2.23 | -0.08 |
|  | 277 | 68.9 | 1.09 | 3.79 | 4.26 | 2.27 | 0.22 |
|  | 279 | 70.4 | 1.01 | 3.68 | 4.44 | 2.18 | -0.73 |
|  | 281 | 71.9 | 1.12 | 5.00 | 4.20 | 2.17 | 0.23 |
|  | 283 | 73.4 | 1.10 | 5.51 | 3.94 | 2.02 | -0.03 |
|  | 285 | 75.0 | 1.54 | 11.52 | 3.06 | 2.47 | 0.12 |
|  | 287 | 76.5 | 1.70 | 13.49 | 2.86 | 2.48 | 0.18 |
|  | 289 | 78.0 | 2.78 | 26.30 | 2.14 | 2.52 | -0.26 |
|  | 291 | 79.5 | 2.92 | 27.87 | 2.10 | 2.59 | 0.00 |
|  | 293 | 84.0 | 11.56 | 130.04 | 1.52 | 5.55 | 0.24 |
|  | 295 | 84.9 | 8.82 | 106.87 | 1.59 | 4.06 | 0.30 |
|  | 297 | 85.7 | 1.91 | 18.31 | 2.59 | 2.11 | 0.30 |
|  | 299 | 86.5 | 0.77 | 2.21 | 4.74 | 1.83 | 0.03 |
|  | 301 | 87.4 | 0.77 | 1.78 | 4.99 | 1.93 | 0.21 |
|  | 303 | 88.2 | 0.80 | 1.90 | 4.61 | 1.95 | 0.09 |
|  | 305 | 89.0 | 0.82 | 1.75 | 4.44 | 1.97 | 0.51 |
|  | 307 | 89.9 | 0.82 | 1.32 | 4.43 | 2.16 | -0.56 |
|  | 309 | 90.7 | 0.81 | 1.27 | 4.44 | 2.07 | 0.04 |
|  | 311 | 91.5 | 0.79 | 1.53 | 4.57 | 2.19 | 0.28 |
|  | 313 | 92.3 | 0.63 | 0.96 | 4.23 | 1.82 | 0.00 |
|  | 315 | 93.2 | 0.88 | 1.44 | 6.16 | 2.35 | 0.12 |
|  | 317 | 94.0 | 0.78 | 1.51 | 4.45 | 2.14 | -0.04 |
|  | 319 | 94.8 | 0.52 | 1.15 | 5.49 | 1.44 | -0.13 |
|  | 321 | 95.7 | 0.61 | 1.34 | 5.25 | 1.66 | 0.14 |
|  | 323 | 97.7 | 0.76 | 1.19 | 3.97 | 2.06 | -0.40 |
|  | 325 | 99.7 | 0.66 | 1.21 | 6.03 | 1.72 | 0.01 |
|  | 327 | 101.8 | 0.71 | 1.22 | 5.11 | 1.76 | 0.06 |
|  | 329 | 103.8 | 0.63 | 1.01 | 4.29 | 1.63 | -0.20 |
|  | 331 | 105.9 | 0.49 | 0.94 | 4.71 | 1.22 | -0.47 |
|  | 333 | 107.9 | 0.72 | 1.15 | 3.49 | 1.75 | -0.14 |
|  | 335 | 109.4 | 1.15 | 1.61 | 5.02 | 2.97 | 0.12 |
|  | 337 | 110.2 | 0.66 | 1.17 | 4.18 | 1.62 | 0.18 |
|  | 339 | 111.0 | 0.77 | 1.30 | 3.94 | 1.91 | -0.06 |
|  | 341 | 111.8 | 1.01 | 1.38 | 3.61 | 2.56 | -0.44 |
|  | 343 | 112.6 | 0.83 | 1.16 | 3.56 | 2.27 | -0.54 |
|  | 345 | 113.4 | 0.94 | 1.35 | 3.90 | 2.69 | -1.07 |
|  | 347 | 114.2 | 0.92 | 1.24 | 4.03 | 2.54 | -0.04 |
|  | 349 | 115.0 | 0.91 | 1.23 | 4.16 | 2.50 | -0.10 |
|  | 351 | 115.8 | 0.89 | 1.24 | 4.70 | 2.52 | -0.20 |
|  | 353 | 118.2 | 0.85 | 1.18 | 4.56 | 2.45 | -0.41 |
|  | 355 | 119.0 | 0.86 | 1.13 | 4.71 | 2.84 | -0.12 |
|  | 361 | 119.8 | 0.81 | 1.13 | 4.37 | 2.67 | -0.26 |
|  | 363 | 120.6 | 0.73 | 1.06 | 4.47 | 2.47 | -0.41 |
|  | 365 | 121.4 | 1.19 | 1.57 | 4.21 | 4.34 | -0.27 |
|  | 367 | 122.2 | 0.91 | 1.23 | 4.55 | 3.18 | -0.65 |
|  | 369 | 123.0 | 0.61 | 1.07 | 4.02 | 2.17 | 0.07 |
|  | 371 | 123.7 | 0.78 | 1.28 | 3.64 | 2.49 | -0.57 |
|  | 373 | 124.1 | 0.75 | 1.29 | 3.92 | 2.19 | -0.82 |
|  | 375 | 124.9 | 0.77 | 1.33 | 3.74 | 2.25 | -0.34 |
|  | 377 | 125.3 | 0.99 | 1.80 | 3.14 | 2.84 | -0.63 |
|  | 379 | 125.6 | 0.92 | 1.70 | 3.06 | 2.54 | -0.19 |
|  | 381 | 126.0 | 1.01 | 2.07 | 3.01 | 2.57 | -0.17 |
|  | 383 | 126.4 | 0.88 | 2.03 | 3.55 | 2.26 | 0.06 |
|  | 385 | 126.8 | 1.11 | 3.34 | 3.77 | 2.02 | 0.07 |
|  | 387 | 127.2 | 1.17 | 4.24 | 4.61 | 2.20 | -0.31 |
|  | 389 | 127.6 | 0.64 | 1.90 | 6.26 | 1.52 | -0.14 |
|  | 391 | 127.9 | 0.66 | 2.50 | 8.77 | 1.20 | 0.24 |
|  | 393 | 128.3 | 0.68 | 2.19 | 8.47 | 1.29 | 0.08 |
|  | 395 | 128.7 | 0.75 | 1.86 | 8.05 | 1.62 | -1.05 |
|  | 397 | 129.1 | 0.76 | 1.65 | 7.18 | 1.64 | -0.05 |
|  | 399 | 129.5 | 0.80 | 1.41 | 7.38 | 1.73 | 0.11 |
|  | 401 | 129.9 | 0.75 | 1.32 | 6.45 | 1.76 | 0.32 |
|  | 403 | 130.3 | 0.89 | 1.72 | 5.91 | 2.03 | 0.01 |
|  | 405 | 131.8 | 0.84 | 1.49 | 7.39 | 1.93 | -0.28 |
|  | 407 | 133.3 | 0.82 | 1.44 | 6.39 | 1.73 | 0.26 |
|  | 409 | 134.7 | 0.80 | 1.48 | 5.83 | 1.76 | 0.30 |
|  | 411 | 136.2 | 0.72 | 1.06 | 6.25 | 1.74 | 0.10 |
|  | 413 | 137.7 | 0.96 | 1.46 | 5.73 | 2.07 | 0.32 |
|  | 415 | 139.2 | 0.80 | 1.18 | 5.69 | 1.85 | 0.69 |
|  | 417 | 140.7 | 0.84 | 1.25 | 5.39 | 1.95 | 0.46 |
|  | 419 | 142.2 | 0.97 | 1.51 | 4.64 | 2.23 | 0.39 |
|  | 421 | 143.7 | 1.06 | 1.52 | 4.63 | 2.42 | 0.24 |
|  | 423 | 145.2 | 1.01 | 1.49 | 5.06 | 2.39 | 0.47 |
|  | 425 | 146.7 | 0.95 | 1.44 | 5.67 | 2.16 | 0.23 |
|  | 427 | 148.3 | 0.93 | 1.45 | 5.59 | 2.13 | 0.58 |
|  | 429 | 149.8 | 0.79 | 1.23 | 6.04 | 1.80 | 0.38 |
|  | 431 | 151.3 | 0.81 | 1.30 | 5.51 | 1.83 | 0.31 |
|  | 433 | 152.9 | 0.76 | 1.21 | 5.93 | 1.75 | 0.55 |
|  | 435 | 154.4 | 0.82 | 1.32 | 5.50 | 1.85 | 0.14 |
|  | 437 | 156.0 | 0.90 | 1.41 | 5.56 | 1.94 | 0.30 |
|  | 439 | 157.5 | 0.89 | 1.47 | 5.84 | 1.92 | 0.38 |
|  | 441 | 159.1 | 0.73 | 1.26 | 6.86 | 1.56 | 0.17 |
|  | 443 | 160.6 | 0.93 | 1.57 | 6.65 | 2.03 | 0.21 |
|  | 445 | 162.1 | 0.78 | 1.32 | 6.67 | 1.66 | 0.47 |
|  | 447 | 163.5 | 0.61 | 1.10 | 7.30 | 1.37 | 0.39 |
|  | 449 | 164.2 | 0.56 | 1.04 | 7.52 | 1.24 | 0.48 |
|  | 451 | 164.9 | 0.70 | 1.17 | 6.99 | 1.54 | 0.39 |
|  | 453 | 165.6 | 0.65 | 1.03 | 7.24 | 0.41 | 0.10 |
|  | 455 | 166.4 | 0.82 | 1.29 | 6.67 | 0.40 | -0.06 |
|  | 457 | 167.1 | 0.78 | 1.15 | 6.52 | 1.15 | -0.15 |
|  | 459 | 167.8 | 0.70 | 0.98 | 6.35 | 1.42 | 0.03 |
|  | 461 | 168.5 | 0.02 | 0.32 | 3.05 | 0.03 | 0.06 |
|  | 463 | 169.3 | 0.80 | 1.15 | 5.59 | 1.45 | 0.00 |
|  | 465 | 170.0 | 1.16 | 1.52 | 5.67 | 2.72 | 0.17 |
|  | 467 | 170.7 | 0.72 | 1.07 | 5.81 | 1.73 | 0.02 |
|  | 469 | 171.5 | 0.82 | 1.18 | 5.74 | 1.98 | 0.02 |
|  | 471 | 172.2 | 0.89 | 1.21 | 5.30 | 2.07 | 0.20 |
|  | 473 | 172.9 | 0.82 | 1.18 | 5.71 | 1.91 | 0.13 |
|  | 475 | 173.6 | 0.76 | 1.10 | 5.32 | 1.81 | -0.44 |
|  | 477 | 174.4 | 0.84 | 1.21 | 5.31 | 1.92 | -0.02 |
|  | 479 | 175.1 | 0.75 | 1.04 | 5.60 | 1.75 | 0.24 |
|  | 481 | 175.8 | 0.83 | 1.19 | 5.04 | 1.89 | 0.11 |
|  | 483 | 176.5 | 0.92 | 1.43 | 5.51 | 2.05 | 0.01 |
|  | 485 | 177.3 | 0.76 | 1.10 | 5.94 | 1.62 | -0.14 |
|  | 487 | 178.0 | 0.68 | 0.96 | 5.79 | 1.41 | -0.01 |

**Supplemental Table 2**

| **sample ID** | **average age (kyr)** | **2σ error**  **(kyr)** |
| --- | --- | --- |
| MV1014-08JC 0-2 cm | 1132 | 78 |
| MV1014-08JC 6-8 cm | 1504 | 91 |
| MV1014-08JC 30-32 cm | 10070 | 138 |
| MV1014-08JC 40-42 cm | 12913 | 190 |
| MV1014-08JC 60-62 cm | 17278 | 329 |
| MV1014-08JC 80-82 cm | 20728 | 450 |
| MV1014-08JC 100-102 cm | 23702 | 476 |
